# SingleMALD: Investigating practice effects in auditory lexical decision

**DOI:** 10.3758/s13428-025-02628-z

**Published:** 2025-04-02

**Authors:** Filip Nenadić, Katarina Bujandrić, Matthew C. Kelley, Benjamin V. Tucker

**Affiliations:** 1 Department of Linguistics, University of Potsdam, Haus 14 (Room II.14.313) Karl-Liebknecht-Straße 24-25, 14476 Potsdam, Germany; 2 Independent researcher, Berlin, Germany; 3 Linguistics Program, Department of English, George Mason University, Fairfax, VA USA; 4 Department of Communication Sciences and Disorders, Northern Arizona University, Flagstaff, AZ USA; 5 Department of Linguistics, University of Alberta, Edmonton, Canada

**Keywords:** Auditory lexical decision, Megastudy, Fully crossed design, Practice effects

## Abstract

We present SingleMALD, a large-scale auditory lexical decision study in English with a fully crossed design. SingleMALD is freely available and includes over 2 million trials in which 40 native speakers of English responded to over 26,000 different words and over 9000 different pseudowords, each in 67 balanced sessions. SingleMALD features a large number of responses per stimulus, but a smaller number of participants, thus complementing the Massive Auditory Lexical Decision (MALD) dataset which features many listeners but fewer responses per stimulus. In the present report, we also use SingleMALD data to explore how extensive testing affects performance in the auditory lexical decision task. SingleMALD participants show signs of favoring speed over accuracy as the sessions unfold. Additionally, we find that the relationship between participant performance and two lexical predictors – word frequency and phonological neighborhood density – changes as sessions unfold, especially for certain lexical predictor values. We note that none of the changes are drastic, indicating that data collected from participants that have been extensively tested is usable, although we recommend accounting for participant experience with the task when performing statistical analyses of the data.

## Introduction

In psycholinguistic megastudies, a very large number of participants respond to a very large number of language items to yield a data set of size and variety unmatched by smaller, targeted experiments (Balota et al., 2012; Keuleers & Balota, 2015). Targeted experiments are often suitable for answering specific questions, but the megastudy approach complements them through its unique advantages. These advantages include higher generalizability of the findings across participants and items, reduced item-sampling bias, higher statistical power, and the possibility of testing complex statistical models (i.e., joint consideration of a larger number of predictors and exploration of continuous and non-linear effects; an advantage particularly important in studies that cannot exert full experimental manipulation of independent variables). Furthermore, megastudies can serve as a benchmark for comparison with the results obtained in targeted experiments or can be used to create virtual/pilot experiments and generate specific hypotheses that may be further tested in targeted experiments. Depending on the behavioral task, megastudies can also be valuable sources of experimental material such as generated pseudowords, item audio recordings, or visual images.

The megastudy approach has now been applied in a number of behavioral tasks, far too numerous to be listed here. The present paper investigates spoken word recognition using the auditory lexical decision task (see Goldinger, 1996; Marslen-Wilson, 1980). In the auditory lexical decision task, listeners are presented with audio recordings of spoken words and pseudowords (phonotactically licit utterances that resemble words, but have no meaning) and are tasked with deciding whether what they heard is a word or not a word as quickly and as accurately as possible. Two direct measures of participant performance can be collected: response accuracy and response latency; if a certain stimulus, participant, or contextual variable predicts or influences performance, researchers assume that the given variable plays a role in the process of spoken word recognition. Megastudies implementing the auditory lexical decision task were conducted in Dutch in the Biggest Auditory Lexical Decision Experiment Yet (BALDEY, Ernestus & Cutler, 2015), in French in the MEGALEX project (note that MEGALEX also collected data for the visual lexical decision task, Ferrand et al., 2018), and in English in two separate studies: the Massive Auditory Lexical Decision (MALD, Tucker et al., 2019) and the Auditory English Lexicon Project (AELP, Goh et al., 2020).

Researchers investigating word processing in the (auditory) lexical decision task aim for a large number of items to capture the variety present in the language and large item power, i.e., many responses per item. Acquiring both simultaneously is difficult as one comes at the cost of the other. Designs favoring many responses per item in which each participant responds to all of the stimuli are usually referred to as fully crossed, while those in which a larger number of participants respond to a subset of stimuli (an application of matrix sampling) are said to be not fully crossed (Keuleers et al., 2010). For example, projects like BALDEY (Ernestus & Cutler, 2015) use a fully crossed design. In BALDEY, the same 20 participants responded to all of the 2780 word stimuli. This design sacrifices variety in individual differences for the sake of higher item power and the ability to compare directly between words. A matrix sampling design was implemented in lexical decision megastudies in the two English auditory lexical decision megastudies.

In AELP (Goh et al., 2020), each of the 10,170 words and 10,170 pseudowords was recorded by six different speakers, one male and one female, for each of the three considered dialects of English. Four hundred and thirty-eight participants provided lexical decisions responding to only one token (i.e., recording version of a given item) throughout their participation in the experiment. The participants responded to 678 words and 678 pseudowords within a single session. Most of the participants completed the maximum possible number of five sessions. The AELP data set contains 25–36 responses per token, or 150–216 responses per item.

MALD (Tucker et al., 2019, v1.1) includes 459,147 responses from 521 participants to 26,793 words and 9592 pseudowords recorded by one speaker of Western Canadian English. Participants completed up to three sessions, each containing 400 word and 400 pseudoword stimuli. Between 6 and 12 responses were recorded for each word. Finally, MEGALEX (Ferrand et al., 2018) tested 101 participants who responded to half of 17,876 computer-synthesized auditory word stimuli and as many synthesized pseudoword stimuli. Although technically implementing a matrix sampling design, MEGALEX still features fairly many trials per listener and fairly many trials per stimulus, positioning itself somewhere between BALDEY and AELP.

### Effects of extensive testing in the lexical decision task

Performing a certain task thousands of times may impact participant performance. Important questions about practice effects arise when using a fully crossed design. Are the effects recorded in fully crossed megastudies comparable to those recorded in matrix-sampled megastudies? If the effects are different, what changes are observed, and can changes in performance across sessions tell us about potential changes that occurred in the word recognition process?

These questions regarding practice effects in lexical decision studies have been raised by Keuleers et al. (2010). Their Dutch Lexicon Project is a database of visual lexical decision responses collected through a fully crossed design. Thirty-nine participants responded to more than 14,000 words and more than 14,000 pseudowords split into 57 blocks. The participants came in multiple sessions, completing a variable number of blocks per session. Keuleers et al. (2010) reported a general trend of response latency decreasing from block to block. This decreasing trend held for both word and pseudoword responses. At the same time, response accuracy for words and pseudowords remained roughly stable throughout the study.

Keuleers et al. (2010) conducted two additional analyses to investigate how the effects of certain lexical predictors change throughout the study. In the first analysis, they compared the nonlinear effects of word frequency, OLD20 (orthographic Levenshtein distance 20; Yarkoni et al., 2008), and word length in the first and last block. In the last block, the frequency effect was weaker, as was the length effect, but the effect of OLD20 was stronger. However, the magnitude of these differences due to practice was reported to be rather small. In the second analysis, the authors examined how the linear effects of these predictors changed across the 57 blocks. They found that the effect of frequency steadily decreased until block 40. After block 40, the effect of frequency was still observed to be large and did not decrease further. The effect of word length was weak overall and had no clear trend across blocks. Finally, the effect of OLD20 was slightly stronger and somewhat increased as participants progressed through blocks.

Keuleers et al. (2010) do not provide specific explanations of the documented practice effects, likely because of the effects’ small size. Instead, the authors conclude that lexical decision data collected through fully crossed designs is valid if the pseudowords are carefully constructed. Pseudoword construction should aim to avoid any systematic differences between words and pseudowords, as participants could implicitly learn these differences throughout the sessions, thus altering the cues they rely on in the lexical decision task.

Hargreaves and Pexman (2012) also performed an exhaustive analysis of practice effects in visual lexical decision using data from the British Lexicon Project (Keuleers et al., 2012). The authors analyzed the response latency of correct responses to word stimuli. As the study progressed through 57 blocks, response latency decreased. Importantly, the effect of various predictors interacted with block number (1–27 versus 28–56), indicating a change in their importance in later versus earlier blocks. The effect of frequency was again smaller with more practice, but the effect of OLD20 was stable and the effects of word length and of average radius of co-occurrence (ARC, a measure of semantic neighborhood density; Shaoul & Westbury, 2010) were only slightly diminished as the study progressed. In a separate analysis, the authors considered a much smaller set of 3,723 words for which they had values for age of acquisition (Cortese & Khanna, 2008; Stadthagen-Gonzalez & Davis, 2006), imageability (Cortese & Fugett, 2004; Stadthagen-Gonzalez & Davis, 2006), and number of senses (operationalized as the number of discrete entries on http://www.wordsmyth.net). They noted that imageability, like frequency, lost some of its effect in later blocks, while the effect of age of acquisition and number of senses remained equal in early versus later blocks.

Hargreaves and Pexman (2012) offer two interpretations for the decreased frequency effect. First, adopting a diffusion model framework described by Dutilh et al. (2009), they suggest that extensive practice led to faster information accumulation and decreased response caution. These factors together restricted the window of time for frequency information to accumulate before a response is made, thus attenuating the frequency effect. Second, because the stimulus set contained a large number of low-frequency words, they argue that participants may have learned over time that frequency is not a useful cue to lexicality. However, the authors do not interpret the practice effects concerning OLD20, word length, and age of acquisition (beyond noting the dissociation between age of acquisition and frequency). Instead, they focus on the trends with regard to semantic characteristics. The authors claim that practice increased participants’ efficiency in making lexical decisions, making semantic processing progressively shallower. This quicker and shallower processing allowed only fast semantic processes to unfold (as indexed by the effect of number of senses or ARC) while attenuating slower semantic processes that reflect sensory experience with a given object (reflected in imageability).

Where auditory lexical decision is concerned, although practice effects are noted by researchers employing fully crossed designs, they are inspected in less detail. In BALDEY, Ernestus and Cutler (2015) found that their 20 participants responded faster as sessions progressed from the first to the tenth session. Participant error rate, in turn, did not change significantly between sessions, but the participants became less accurate as a session progressed (within session). Similarly, Ferrand et al. (2018) describes the practice effects registered in both the visual and the auditory lexical decision task. In the auditory modality, their 101 participants show a reduction in response latency, but no substantial change in response accuracy as they participate in more and more of the 100 study blocks. Neither the BALDEY nor MEGALEX authors report testing for an interaction between session or block number and other pertinent predictors such as word frequency.

### The present study

To the best of our knowledge, there is no fully crossed auditory lexical decision megastudy for the English language. The first goal of the present study is to remedy this gap. We present SingleMALD, a freely available fully crossed branch of the Massive Auditory Lexical Decision project (Tucker et al., 2019). The SingleMALD dataset includes responses from 40 listeners to, approximately, 26,700 different MALD words and 9600 different MALD pseudowords collected in 67 twenty-five-minute sessions held across 35 participation days. In sum, the SingleMALD dataset includes data from 2,139,766 trials, which is 4.66 times more than the current version of MALD (v1.1; 459,147 trials). MALD includes responses to a large number of items from a large number of participants, but the number of responses per particular item is not very high. Consequently, MALD performs well in capturing the general effects of stimuli characteristics (e.g., phonological neighborhood density or word frequency), while also offering a higher degree of confidence that these effects generalize across stimuli and listeners that have the same demographic characteristics as MALD listeners. However, given that each stimulus is encountered by only a small subset of MALD listeners, the number of responses per item in MALD is not large; this is especially the case for word stimuli. In that context, SingleMALD complements MALD. SingleMALD likewise contains responses to many stimuli (the same as in MALD) but offers a substantially higher item power compared to the standard MALD database as all SingleMALD participants responded to all stimuli. In turn, SingleMALD has decreased listener variability compared to MALD and features data from listeners who performed the auditory lexical decision task extensively. We encourage researchers to carefully consider these advantages and disadvantages when selecting which dataset to use. We also note that given that the same session structure and stimuli were used in MALD and SingleMALD, the datasets from these two studies may also be merged into a single dataset.

The second goal of the present study is to investigate the effects of repeated testing in the auditory lexical decision task when recordings of a single speaker are used as stimuli. It is important to test whether the changes in effects noted in fully crossed visual lexical decision studies are the same when the stimuli are presented in the auditory modality. There is much more variability between different utterances than between different written fonts (which is not usually varied in a visual lexical decision task). SingleMALD uses recordings of a single person, meaning that the listeners can become used to the fine phonetic detail in the speaker’s voice during the course of the study. Listeners change their performance as they adapt to the vocal characteristics of a particular speaker or groups of speakers (see, e.g., Fenn et al., 2003; Kleinschmidt & Jaeger, 2015; Kraljic & Samuel, 2005; 2006; Magnuson & Nusbaum, 2007; Magnuson et al., 2021; Norris et al., 2003; Theodore & Monto, 2019). Still, many of these studies show that the benefits of getting used to a particular speaker or dialect are observed even with brief exposure. It remains to be seen what the extent of these practice effects is and when they start emerging in an extended auditory lexical decision experiment that uses isolated stimuli as learning material for the listener. Also, we still do not know how familiarization with the talker and the task impacts participant strategy, i.e., affects the predictive value of salient predictors such as word frequency.

We also note that the task demands are somewhat different in SingleMALD in comparison to previous studies. SingleMALD is almost ten times longer than BALDEY (Ernestus & Cutler, 2015) in terms of the number of trials a single participant completes. MEGALEX (Ferrand et al., 2018) and AELP (Goh et al., 2020) used the same procedure applied in the Dutch Lexicon Project (Keuleers et al., 2010), where the participants received feedback about their performance and had an incentive to keep their accuracy above 80% in order to earn financial compensation for their time and be retained in the study. In SingleMALD, the participants were instructed to respond as quickly and as accurately as possible, but they did not receive any feedback nor was their compensation impacted by their performance. In MEGALEX Ferrand et al. (2018), listeners responded to computer-synthesized speech rather than recorded speech as in SingleMALD and the other mentioned auditory megastudies (but note that the effects of training with synthesized speech are also documented; see Schwab et al., 1985). The pronunciations in AELP appear to us to be more careful than the MALD recordings, as the MALD speaker was instructed to produce the words and pseudowords as naturally as possible. These differences in task demands could lead to differences in participant strategies and affect their performance as the study unfolds, making direct comparisons between different fully crossed auditory lexical decision studies difficult.

## Methods

The methods reported in this paper follow closely the methods reported in Tucker et al. (2019). In the present paper, we have simplified the description while trying to provide a sufficient description of the methods. In this description, we have focused on aspects that are different between this report and the previous work.

### Items

We used the same items as those reported in Tucker et al. (2019). For item selection and creation, we attempted to select a lexicon of words and pseudowords that generalized across the spoken English lexicon. This process resulted in 28,510 words. The word list included mono- and multi-morphemic words, inflected and derived forms, function and content words, compound words, and all parts of speech apart from proper nouns and coordinating conjunctions. Pseudowords for the project were generated from the 28,510 words using the software package *Wuggy* (Keuleers & Brysbaert, 2010) by replacing one-third of the subsyllabic constituents of each word. This resulted in the creation of English-like pseudowords which were both simplex and complex, for example  ,  ,  ,  (for a detailed analysis of pseudoword characteristics and their processing see Kelley & Tucker, 2022a; Tucker et al., 2019). We randomly selected 11,400 pseudowords to optimize the time spent in recording and preparation.

One 28-year-old Western Canadian male undergraduate student majoring in linguistics was recorded. On average, recording sessions lasted 2 h. The recording sessions always occurred on weekday afternoons and were not performed if the speaker was in any way sick. The speaker was allowed to take as many breaks as they needed during the recording sessions. The speaker was asked to read the words “as naturally as possible” and to read the pseudowords as though they were words. Words were presented one at a time using the standard spelling and pseudowords were presented one at a time using a phonemic transcription. Words were recorded in 15 recording sessions and pseudowords in ten recording sessions. Words were produced once each and pseudowords were produced at least three times so that the most fluent production could be selected. An experimenter monitored the recording for errors or disfluencies, noting items that required re-recording.

We selected 26,800 word and 9600 pseudoword recordings as experimental stimuli. The stimuli were extracted from the session recordings into individual sound files at zero-crossings and each individual sound file was normalized to 70-dB mean intensity using Praat’s (Boersma & Weenink, 2019) Scale intensity function. Praat TextGrid files were created containing time-aligned phone-level transcriptions for both word and pseudoword stimuli. The word stimuli were divided into 67 lists of 400 words and the pseudoword stimuli were divided into 24 lists of 400 pseudowords. Each word list was then paired in a rotating fashion with two different pseudoword lists, creating 134 800-item *Sessions*.

### Listeners

Fifty-two native monolingual speakers of Western Canadian English were recruited from the University of Alberta. Each participant received compensation in form of 10 CAD for each hour they participated and an additional 100 CAD upon completion of all the experimental sessions. Eleven participants did not complete the full experiment (dropping out after 1 to 41 sessions) and their data is not considered in the present report. One additional participant was excluded due to low performance and not following experimental procedures. The remaining 40 listeners were 21 women and 19 men, aged 18–29 ($M=22.70$, $SD=2.62$).

### Experimental procedure

For each participant, the first day of participation included a hearing evaluation, background questionnaire, and the first of 67 SingleMALD sessions. We used a Maico MA25 audiometer and presented a 20 dB SPL pure tone to each ear at 500 Hz, 1000 Hz, 2000 Hz, and 4000 Hz to perform the hearing evaluation. The background questionnaire focused on experience with dialects of English or foreign languages and its purpose was mainly to ascertain that the participant met the criteria for participation in the study. The collected information is part of the final SingleMALD dataset. Lastly, the participants completed their first SingleMALD session.

Listeners were then scheduled to come to the lab three, four, or five times a week. Every day of their participation they completed two experimental sessions. Listeners were presented with 400 words and 400 pseudowords for each experimental session. One such session lasted between 20 and 25 min. Most participants participated in 67 sessions, responding to each word item set once, but encountering some pseudoword item sets more than once – most often three times. The order in which they completed the item lists was different across participants. Each participant was randomly assigned a list number (1 to 67) and direction of completion (ascending or descending). For example, subject 190128 started with list 44 and went in ascending order (i.e., 44, 45, [..], 67, 1, [..], 43), while subject 190079 started with list 64 and completed the lists in descending order (64, 63, [..], 1, 67, 66, 65). If a participant accidentally repeated a list, only the first instance was retained. If a list was accidentally skipped or if there were technical issues with data collection, the list was repeated after all other lists were completed.

The experiment was conducted in a sound-attenuated booth using E-Prime 2.0 Professional (Schneider et al., 2002) with a Serial Response Box (Psychology Software Tools, Inc) and MB Quart QP805 headphones calibrated with a 1-kHz tone to a level of 81±1 dB (EXTECH 407750 sound level meter with a 2cc-coupler which simulates the resonant frequencies of the ear canal).

The auditory lexical decision task session began with participants registering their participant number, selecting a session number, and responding to a short set of background questions (the background questions were retained to maintain similarity with the procedure that was used with non-fully crossed MALD participants). Listeners were then instructed to decide whether a given item was a word of English or not. They were asked to press a button with their dominant hand to select a “word” response and with their non-dominant hand to select “not a word” response. Each trial had the following structure: (1) a 500-ms fixation mark was presented on the screen, (2) the audio stimulus was presented over the headphones and (3) the participants could make their response on the button box from the auditory stimulus onset. As a result, responding before stimulus offset was possible. After the participant responded or 3000 ms elapsed from stimulus onset, the experiment would progress to the next trial without any feedback provided to the participant.

## Dataframe

The SingleMALD dataframe follows the same structure as the MALD dataframe, which enables merging and direct comparison. The first 56 variables of the SingleMALD dataframe are the same as in the MALD v1.1 dataframe (see Tucker et al., 2019). However, SingleMALD also includes ten new variables described below.

- RunDate. The date of the experimental session.
- RunTime. The time when the experiment was started.
- WordACCRate. The accuracy rate over word items for a particular session.
- PwordACCRate. The accuracy rate over pseudoword items for a particular session.
- NthDay. The number of consecutive days of testing for the listener thus far in the experiment, counting only valid participation days.
- NthSessionOfDay. The number of sessions in which the listener participated thus far in the day, including the present session, counting only valid sessions.
- NthSession. The number of sessions in which the listener participated thus far in the experiment, including the present session, counting only valid sessions.
- DaysSincePrevSession. The number or days since the previous session for that listener, counting only valid sessions. This variable is set to 0 for the first session.
- WeekDay. The day of the week when the experimental session took place.
- NthPseudowordSet. The number of times the listener was exposed to the current pseudoword set, including the present session, counting only valid sessions.

The SingleMALD dataframe includes 2,139,766 trials from 40 listeners in 2675 sessions. Data collection included some minor issues. We only document the issues here, as the SingleMALD data set features them. In the analysis of practice effects reported in the present manuscript, we address these issues in the data preparation procedure, but researchers using SingleMALD are welcome to administer their own data preparation procedures.

By design, all listeners should have completed 67 sessions for a total of 2680 sessions. However, one participant completed 66 usable sessions and two participants completed 65 usable sessions only. Furthermore, small data issues were present at the stimulus level. The SingleMALD data was collected partly concurrently with the MALD data, so it suffers from the same technical issues. The Windows environment is case-insensitive, leading to naming conflicts for eight word/pseudoword pairs whose WAV files were spelled with the same letters using Arpabet conventions (these were words “brown”, “flaws”, “flows”, “gray”, “owl”, “pays”, “says”, “shawl”; and pseudowords  ,  ,  ,  ,  ,  ,  ,  , respectively). In all cases, the word was presented instead of the pseudoword, meaning that some experimental runs have slightly more than 400 words and slightly fewer than 400 pseudowords, as well as that these eight words have more responses than other words.

Additionally, seven words (“hamstring”, “automaton”, “accomplishes”, “calling”, “exhalation”, and “liter”) were excluded from the dataset due to issues with their audio recordings, leading to a loss of 239 trials (0.011%). The WordRunLength variable indicating the number of consecutive trials of the same kind of stimulus was calculated so that these seven items always ended the run (i.e., they were counted neither as words nor pseudowords). Measures of by-session performance such as session accuracy rate or mean session response latency were calculated with these seven items excluded.

Besides small data loss, we note occasional discrepancies related to the expected structure of participation in the experiment. First, we intended to test listeners regularly, every week. The variable DaysSincePrevSession reflects this, as 96.04% sessions were held within four or fewer days since the previous session by the same listener. In some cases, participant illness or public holidays further extended this period, so 3.70% of sessions were held between 5 and 11 days since the last session. However, in rare cases, the participants were unavailable for an extended period of time: in seven sessions (0.26%) the number of days since the previous session for that listener ranges between 12 and 23 days.

Second, we intended for the participants to complete two sessions each day with a short break of up to several minutes between them (with the exception of the first day of participation when one session was held and perhaps the last, if only one session remained). However, one listener once completed three sessions in a single participation day, while another listener completed three sessions in a single participation day three times and four sessions in a single participation day two times. There are also seven occurrences (0.26%) where the break was longer, with the delay between the start times of the two sessions being between 1 and 4 h.

Third, SingleMALD participants were supposed to only utilize Session numbers 1 to 67 when initiating an experimental session. Session numbers 68 to 134 repeat one of the 67 word sets, but paired with a different pseudoword set. In five sessions (0.19%), a participant erroneously imputed a number ranging from 68 to 134 without previously being exposed to the word set associated with that Session number. Because we did not want our listeners to respond to the same word set twice, we decided to retain these sessions, accepting that the word set was paired with a different pseudoword set than for the other SingleMALD listeners. This error also affected the number of times a particular listener was exposed to a particular pseudoword set (variable NthPseudowordSet). By design, each listener was supposed to be presented with the same pseudoword set two or three times (depending on the number of word sets 1 to 67 that the particular pseudoword set “a” to “x” was paired with), but in one case a single participant responded to the same pseudoword set – always paired with a different word set – four times.

Lastly, the design did not feature an explicit requirement to maintain a certain degree of accuracy or speed across sessions, nor was feedback provided to the participants. We therefore note a number of sessions where listener performance was low. In our practice effects analyses in the present paper, we take overall session accuracy (ACCRate) below 60% as the threshold for exclusion. With this criterion, there are 138 (5.16%) sessions with low performance, 123 of which come from the same four participants. We keep these sessions in the SingleMALD dataset as the independent user may have other thresholds that they wish to apply or may be interested in when and for which participants these sessions occur.

Additional specific information about data imperfections can be found in the supplementary materials. For each participant, we also include figures of per-session response latency distributions and response accuracy.

## Practice effects analysis and results

**Fig. 1:**
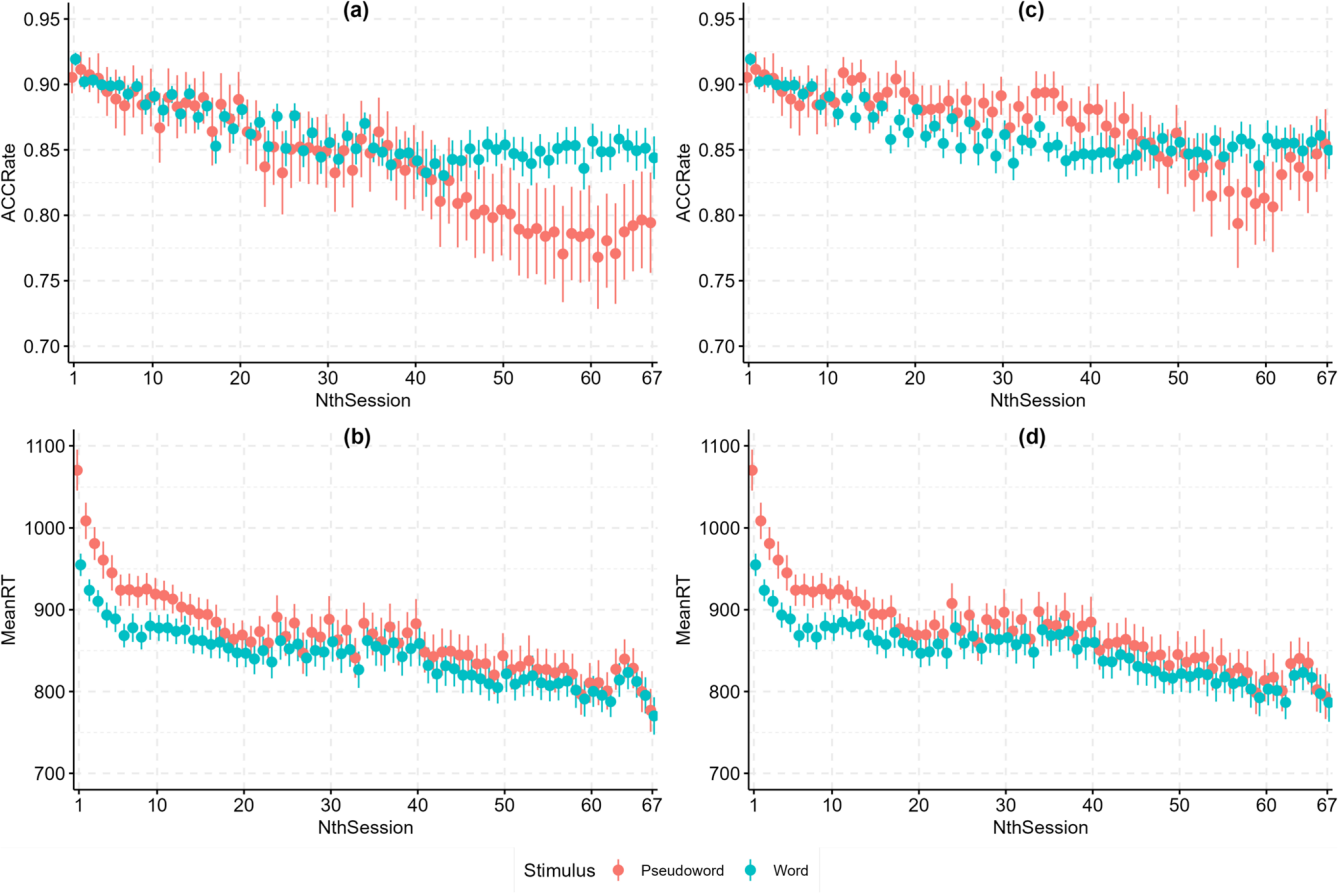
Average participant performance (*y*-axis) as they progress through experimental sessions (*x*-axis) is presented for pseudowords (*red*) and words (*blue*). Plots **a** and **b** given on the left present the data before data exclusion, while plots **c** and **d** given on the right present the data after data exclusion described in the text. Mean response accuracy is presented in the top plots (**a** and **c**) and mean response latency is presented in the bottom plots (**b** and **d**). The *error bar*s represent standard errors of the mean. Note that the *y*-axis range is restricted in all four plots

### Descriptive statistics

Initial observations of averaged participant performance showed a steady reduction in response latency and response accuracy as the study progressed, which is shown in plots (a) and (b) in Fig. 1. In both cases we see a larger decrease in earlier sessions, while average participant performance becomes more stable after approximately 40 sessions. An exception is pseudoword accuracy which decreases substantially past NthSession 40. This is in part because participants started favoring the “word” response, thus reducing their accuracy to pseudowords. For example, the overall percent of “word” responses in NthSession 5 was 50.24% compared to 53.55% in NthSession 60.

The reduction in response accuracy to pseudowords is primarily due to very low accuracy rates recorded in certain sessions. As mentioned in the section detailing the SingleMALD dataframe, there are 138 (5.16%) sessions with overall response accuracy below 60%, 123 (89.13%) of which come from the same four participants. Plots (c) and (d) in Fig. 1 show the changes in participant performance across repeated sessions when these particular participants and sessions are excluded. A comparison of plots (b) and (d) indicates that the overall trend in response latency remains similar (the grand mean changed by 10 ms). A substantial difference can be observed when plots (a) and (c) are compared, especially when pseudoword accuracy is concerned. In plot (c), pseudoword accuracy matches and even surpasses word accuracy approximately between NthSession 15 and 45. In later sessions, pseudoword accuracy is reduced, but not as severely reduced as in plot (a).

In earlier sessions, the participants are conservative about which stimuli they accept as a word and correctly discard pseudoword stimuli, while their response accuracy to words may be limited by their word knowledge. As they progress through the experiment, however, they sacrifice some of their accuracy in favor of speed and start slightly favoring the “word” response. The increased error rate in responses to pseudowords in later sessions of the experiment may also be in part due to pseudoword list repetition. By session 40 and 50, the participants will have already heard most pseudoword recordings at least once or twice and the repeated exposure may cause them to think that they are actual words. Consequently, after approximately NthSession 40, response accuracy to pseudowords decreases, while response accuracy to words slightly increases.

### Generalized additive mixed-effects models

We now turn to investigating how the impact of certain variables on participant performance changes as the experimental sessions unfold. To that end, we use packages mgcv (Wood, 2011) and itsadug (van Rij et al., 2022) in R (R Core Team, 2023) to create and visualize generalized additive mixed-effects models (GAMMs; for examples and recommendations for implementation, see Baayen & Linke, 2020; van Rij et al., 2020; Wieling, 2018). GAMMs can model non-linear effects, making them very useful for exploring changes in participant performance over a larger number of sessions. For the following analyses, we further cleaned our data by (1) excluding eight sessions which were the third or the fourth session in a day for a particular participant, (2) excluding five sessions in which the participant used a Session number ranging from 68-134 (i.e., used a word and pseudoword set combination not seen by other participants), (3) excluding one session where the participant was presented with the same pseudoword set for the fourth time, and (4) excluding trials with response latency lower than 200 ms as unrealistic. We explore how participant response accuracy and response latency change across sessions. Additionally, we explore how the relationship between participant performance and a measure of word length, word form similarity to other words, and word frequency change across sessions.

#### Response accuracy

The dataset used to predict participant response accuracy included 948,626 word trials (86.86% correct) recorded in 2378 sessions from 36 participants. The dependent variable was binomial – the participant responded to the trial either correctly or incorrectly. The statistical model included random intercepts for subject (Subject) and random smooths of trial number (Trial) and NthSession by Subject. Random effects related to specific words were not included because computational time becomes infeasible, but we did include random intercepts per word set presented (List).

The model also included the parametric effects of NthSes-sionOfDay and NthPseudowordSet, and smoothed effects of NthSession, trial number within session (Trial) split by NthSessionOfDay (i.e., an interaction between Trial and NthSessionOfDay), and the number of consecutive trials in which a word stimulus was presented including the present trial (WordRunLength). Besides these situational variables, the model also included logged frequency from the Corpus of Contemporary American English (logFreqCOCA; Davies, 2009), duration in ms (Duration), and phonological neighborhood density (PhonND; number of words that are one phone edit away from the target word). Crucially, the model also included four tensor product interactions between Trial (again split by NthSessionOfDay), Duration, PhonND, and logFreqCOCA on the one hand and NthSession on the other. A tensor product interaction term (created using the ti() function) does not include the main effects which are already separately included in the model; these interactions inform us about the potential change of a particular predictor’s effect as the participant completes more and more sessions.

**Fig. 2:**
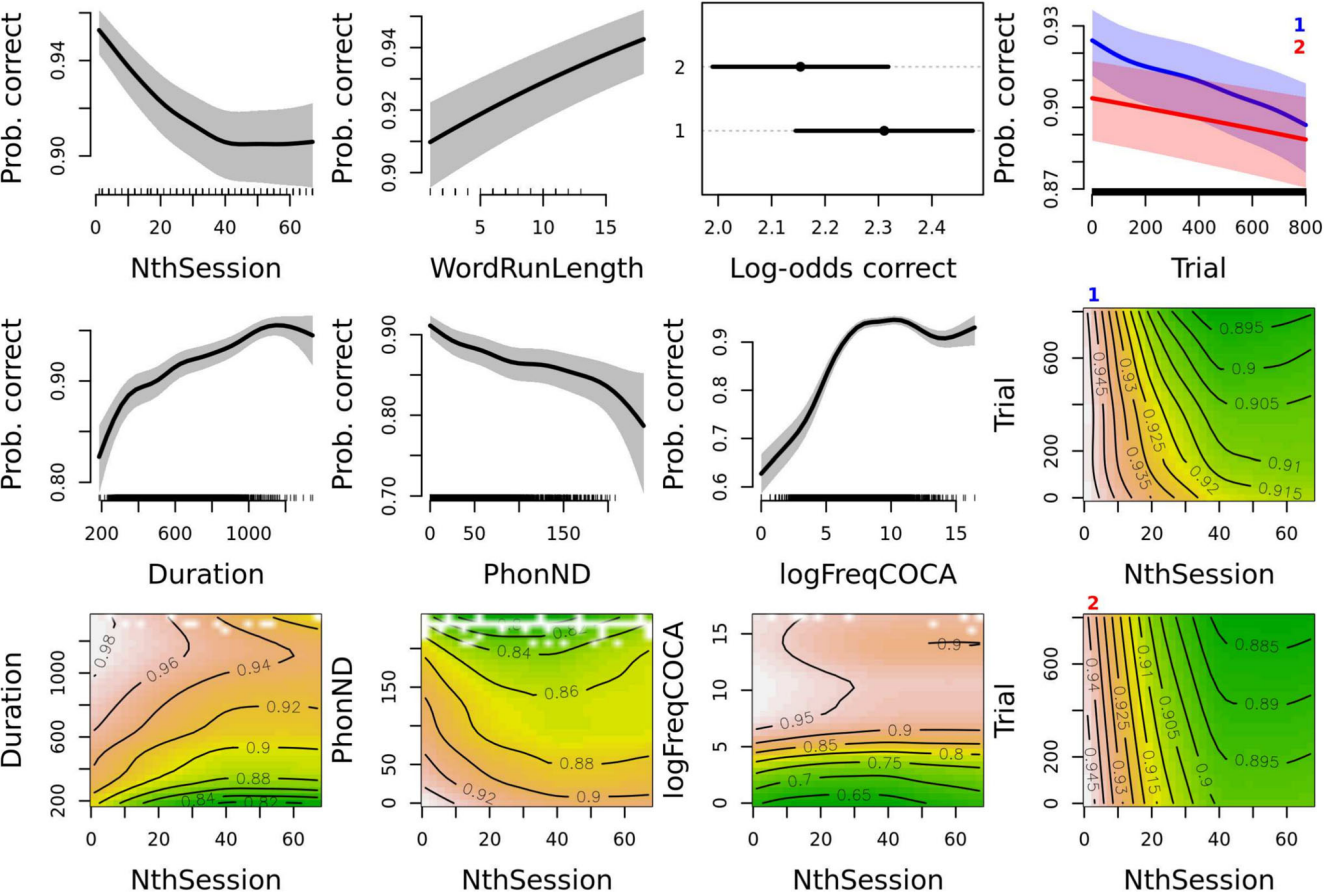
Effects plots of variables for the GAM model fit for response accuracy in all sessions. The plotted estimate is probability of a correct response occurring back-transformed from log-odds. This estimate is plotted on the *y*-axis in main effect plots and on the *z*-axis (color scheme) in interaction plots. The relevant predictors are shown on the *x*-axis for the main effect plots and on the *x*- and *y*-axes for the interaction plots. The exception to these rules is plot three in the first row. This plot shows the parametric effect of NthSessionOfDay where the back-transformation is not performed, while the estimate is plotted on the *x*-axis and the predictor on the *y*-axis. Additionally, the effect of Trial across NthSession is presented separately for the first (*marked with a blue number 1*) and the second (*marked with a red number 2*) NthSessionOfDay. In the interaction plots, higher estimates are associated with *brown color* and lower estimates with *green color*, with the contour lines delineating areas of equal estimated probability values. Note that the range of the presented estimated probability values is adjusted per plot (not all *y*-axis ranges in smooth plots and *z*-axis ranges in interaction plots are equal). Detailed plot interpretation is given in the text

The variable NthPseudowordSet was excluded from the model as it did not significantly improve model fit. All other variables were retained and the model including them is presented in Table 1. The effects in a generalized additive mixed-effects model are best assessed by observing effect plots (Fig. 2). We start by observing the plots in the first row of Fig. 2. We see that the probability of a correct response decreases until NthSession 40, after which the effect dissipates and turns into a flat line. In other words, the participants become somewhat less accurate as they progress through the sessions, likely sacrificing accuracy for speed, but at a certain point the decrease in accuracy levels out; this is at least the case with retained participants and sessions. The smoothed effect of WordRunLength appears largely linear (this is supported by the ‘edf’ value being close to 1 in the model summary presented in Table 1) – more consecutive ‘word’ stimuli lead to higher accuracy when responding to these stimuli. We can also see that the participants were more accurate in their first session on any given day of testing, and that they become somewhat less accurate as they progress through trials of a session: the plot in the upper right corner of Fig. 2 shows the smoothed effect of Trial separately for the first (blue) and the second (red) NthSessionOfDay. The slope of worsening accuracy is larger in the first NthSessionOfDay.

The effects of the situational predictors presented in the first row are smaller than the effects of predictors related to stimulus characteristics presented in the first three plots of the second row of Fig. 2; note that the *y*-axes on plots in Fig. 2 are not all set to the same value range. We see that longer word stimuli have a higher probability of being correctly classified as words by the listener. Words with higher phonological neighborhood density (PhonND) values have a higher probability of being responded to incorrectly. Lastly, higher-frequency words have a substantially larger probability of receiving a correct response. However, once a certain level of logFreqCOCA is reached, the effect tapers off and ceases to provide further benefit.

We now turn to how the effects of Trial, Duration, PhonND, and logFreqCOCA change as the participant progresses through more and more sessions. In Fig. 2, the plots showing the non-linear interactions between NthSession and these predictors are placed directly below the smoothed effect of the predictor in question. The estimated probability of a correct response is represented by different colors reminiscent of a topographic map: lower probability values are associated with green (valleys) and higher probability values are associated with orange and brown (hills and mountains). The interpretation of the plot is further facilitated by contour lines delineating areas of equal estimated probability values. We see that the effect of Duration (bottom left plot) becomes stronger in later sessions, as participant accuracy decreases in shorter stimuli.

In later sessions, participants likely respond briefly after word offset without pausing to think, even if they are uncertain about the lexicality of the stimulus; this strategy is especially detrimental in shorter stimuli. The effect of PhonND (plot two of row three) does not change much across sessions, except that in early-to-middle sessions the contours more clearly distinguish between words with middle-high to very high PhonND. This nuanced effect of larger values of PhonND is largely lost in later sessions. The effect of logFreqCOCA (plot three of row three) appears to be stable across sessions, except perhaps after approximately NthSession 20 where we notice a slight decline in accuracy for highest frequency words (contour line with the 0.95 value is lost). Lastly, the interaction between NthSession and Trial is shown on the fourth plot in row two (first NthSessionOfDay) and in row three (second NthSessionOfDay). The effect of Trial is fairly negligible at first, being overshadowed by NthSession. In other words, participants are fairly consistent within a session early on and after approximately NthSession 20 to 30 in the first NthSessionOfDay, and approximately NthSession 40 in the second NthSessionOfDay the effect of Trial becomes more apparent, as participants are less accurate in later trials within a given session.

Although rich in information, the previously presented plots can be difficult to assess – the nuanced information makes it more difficult to offer clear-cut conclusions and the strong effect of NthSession can overshadow the effects of our variables of interest. Another way to represent changes in participant performance across sessions is to select and directly compare performance in a number of sessions that occurred early and a number of sessions that occurred late in the process of participation. We selected 16 early sessions (NthSession 4 to 19) and 16 late sessions (NthSession 49 to 64), avoiding the initial two and the final 2 days of participation. We then fit similar generalized additive mixed-effects models, replacing NthSession with a categorical distinction between early vs. late sessions (NthSessionCat). Additionally, we did not consider NthPseudowordSet variable as it proved to be insignificant in the previously described model and because it largely overlapped with NthSessionCat. The results of the model are given in Table 2.

**Fig. 3:**
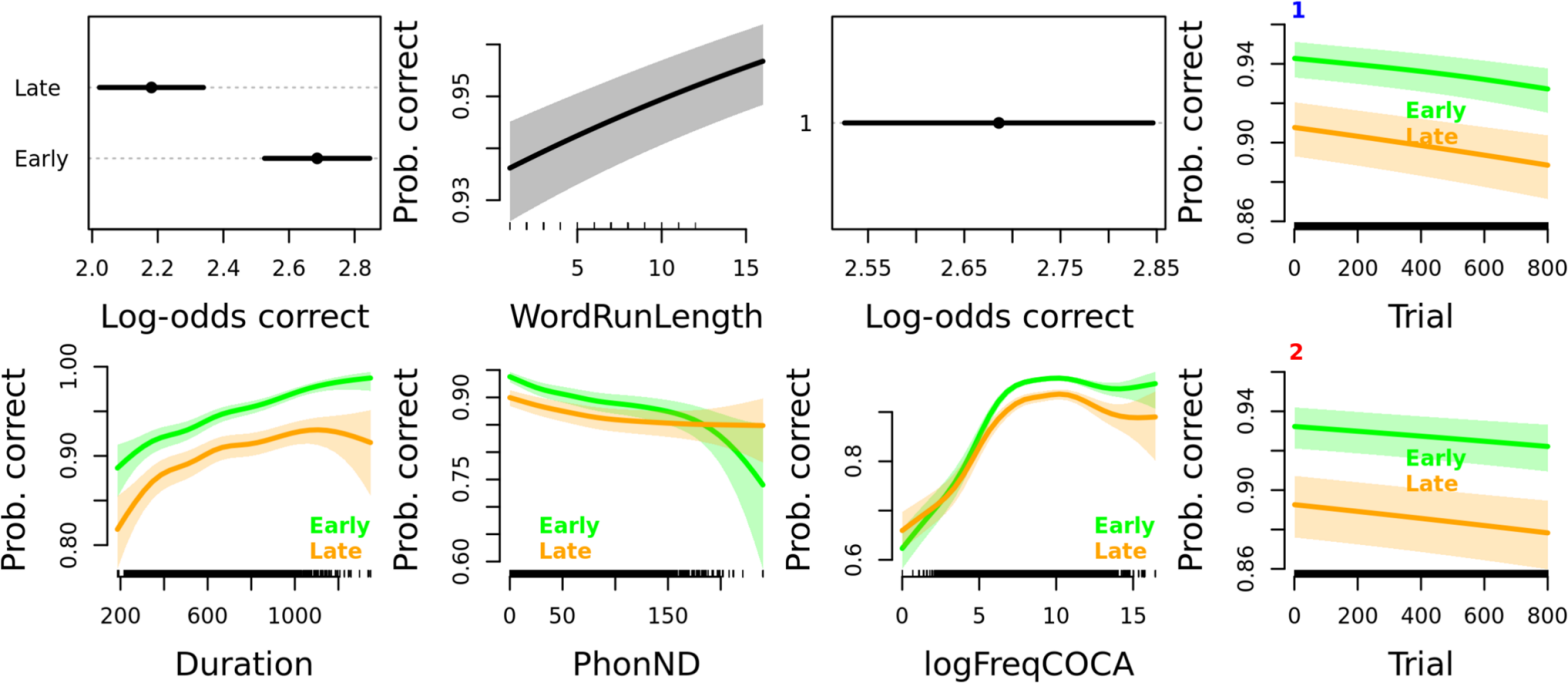
Effects plots of variables for the GAM model fit for response accuracy in early versus late sessions. In parametric effect plots (plot one of row one showing the effect of NthSessionCat and plot three of row one showing the effect of NthSessionOfDay), the estimates plotted on the *x*-axis are log-odds, while the predictor levels are given on the *y*-axis. All other plots are smooth main effect plots, in which the estimate plotted on the *y*-axis is probability of a correct response occurring back-transformed from log-odds, while the continuous predictor is given on the *x*-axis. Data from early sessions is given in *green* and data from late sessions is given in *orange*. The main effects of Trial are presented in the fourth column, separately for the first (*marked with a blue number 1*) and the second (*marked with a red number 2*) NthSessionOfDay. Note that the range of the presented estimated probability values is adjusted per plot (not all *y*-axis ranges in main effect plots are equal), except in the case of the effects of Trial, as the two plots in column four are set to the same *y*-axis value range. Detailed plot interpretation is given in the text

Again, we rely on plots to facilitate interpretation. The first plot in the first row of Fig. 3 shows that the participants are more accurate in earlier sessions. The effect of the control predictor WordRunLength remains the same as in the previous model, with more repetitions of ‘word’ stimuli being associated with a higher chance of their correct classification. The effect of NthSessionOfDay (third plot of row one) is likewise the same as in the model that included all sessions, as the participants are more accurate in the first session of any given day.

The smoothed effect of Trial is presented separately for the first NthSessionOfDay (plot four of row one) and for the second NthSessionOfDay (plot four of row two) and separately for early (green) versus late (orange) sessions. In general, the participants become less accurate as they progress through the session. The second row of Fig. 3 shows the smoothed effects of Duration, PhonND, and logFreqCOCA. The effect of Duration is largely linear in early sessions, but in later sessions there is a steeper slope for the shortest stimuli, as participants likely offer a response immediately upon signal offset, without allowing additional time for processing if they are uncertain of stimulus lexicality. Additionally, the accuracy does not increase further for the longest stimuli; the participants likely stop listening until signal offset for the longest stimuli, favoring speed over accuracy. The clearest change in the effect PhonND has on participant accuracy in late versus early sessions is observed for stimuli with the highest values of PhonND. Whereas high PhonND stimuli were often incorrectly classified in the early sessions, listeners are not affected by this distinction in later sessions. However, we do note that these estimates are based on a smaller number of unique words and individual data points (note the rug on the *x*-axis) and may be unreliable. Lastly, the shape of the smoothed effect of logFreqCOCA appears to be very similar in early and later sessions. Yet, there are differences in the frequency effect in early versus late sessions observed in higher-frequency words. Participant accuracy is reduced in later sessions, but for mid-to-high-frequency words only.

#### Response latency

The statistical model used for response latency was the same as the model used for accuracy, except that the dependent variable in the model was response latency (RT). Following the guidelines in Baayen and Milin (2010), response latency was transformed using the following formula: $-1000/RT$. This transformation led to the distribution of residuals better approximating a normal distribution when compared to raw RT or log-transformed RT. We will refer to this variable as transformed response latency (tRT). We only considered correct responses to word stimuli, so the final dataset included 824,017 trials recorded in 2378 sessions from 36 participants. The results of the model calculated across all sessions are presented in Table 3.

**Fig. 4:**
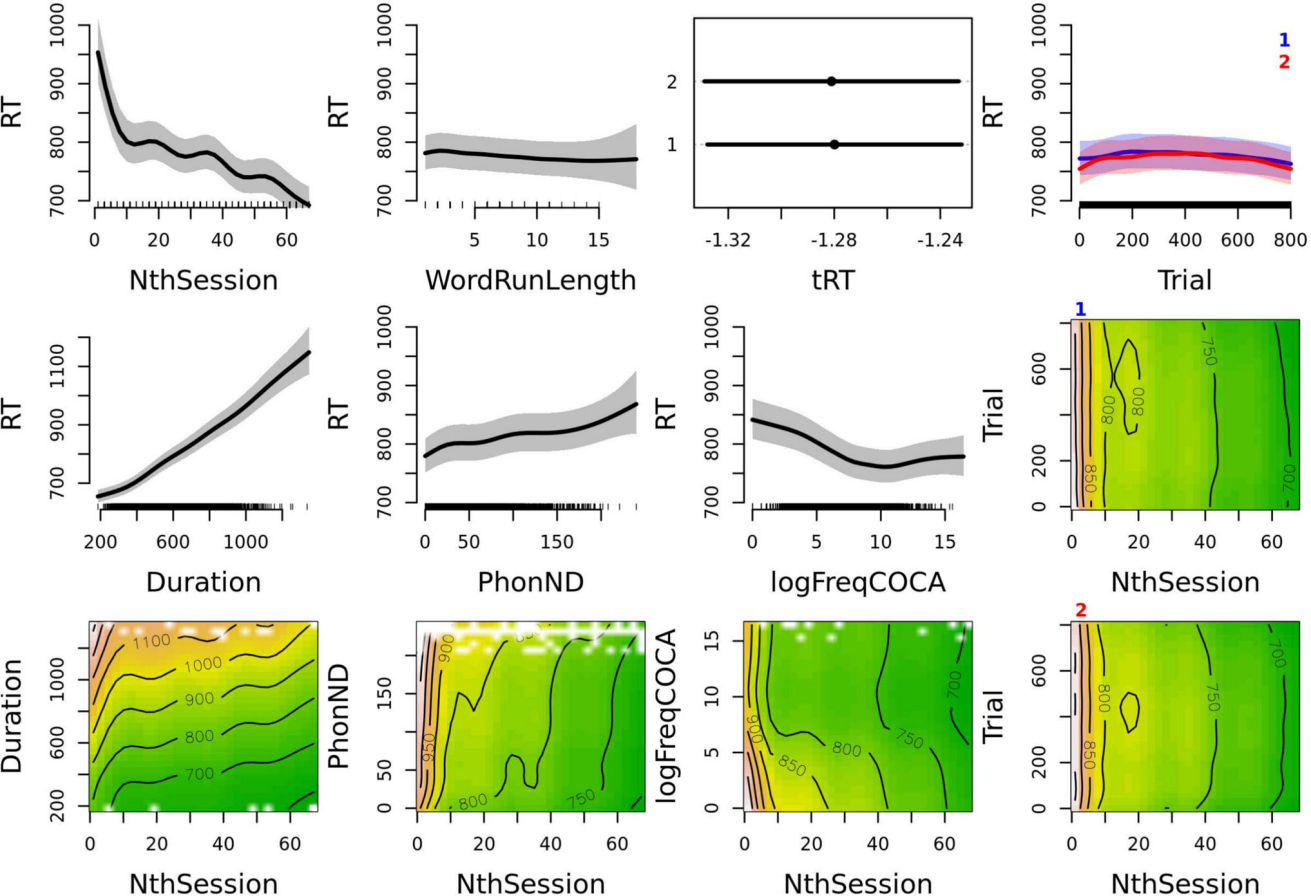
Effects plots of variables for the GAM model fit for response latency in all sessions. The plotted estimate is back-transformed response latency (RT). Response latency is plotted on the *y*-axis in main effect plots and on the *z*-axis (*color scheme*) on interaction plots. The relevant predictors are shown on the *x*-axis for the main effect plots and on the *x*- and *y*-axes for the interaction plots. The exception to these rules is plot three of row one showing the parametric effect of NthSessionOfDay where the transformation is not performed, while the transformed response latency is plotted on the *x*-axis (tRT) and the predictor on the *y*-axis. Additionally, the effect of Trial across NthSession is presented separately for the first (*marked with a blue number 1*) and the second (*marked with a red number 2*) NthSessionOfDay. In the interaction plots, longer RTs are associated with *brown color* and shorter RTs with *green color*, with the contour lines delineating areas of equal RT values. Note that the response latency range is fixed for all smooth effects plots except for the very strong effect of Duration. Response latency ranges are adjusted per plot in the interaction plots. Detailed plot interpretation is given in the text

The participants rapidly decrease their response latency in the first ten sessions (plot one of row one in Fig. 4). Response latency continues to decrease in the following sessions as well, but at a smaller rate. The second plot of row one in Fig. 4 shows a humble linear effect of WordRunLength on response latency with more repetitions of ‘word’ stimuli being associated with somewhat shorter response latency. The effect of NthSessionOfDay is likewise small, with response latency being shorter in the second session of any given day. The last plot in the first row of Fig. 4 shows the effect of Trial split by NthSessionOfDay. At first, the participants slow down slightly and afterwards get faster as they progress through the session.

The first plot in the second row of Fig. 4 shows the effect of Duration; unlike other smooth effects plots, the effect of Duration had to be set on a wider *y*-axis range. As expected, longer stimuli require more time to process and are associated with longer response latency. Higher PhonND is likewise associated with longer response latency. The effect of logFreqCOCA is non-linear: higher frequency words are associated with shorter response latency, but the increase in frequency ceases to be facilitatory at around 8-9 logFreqCOCA. Response latency in trials presenting extremely frequent words is even somewhat longer than in trials presenting very frequent words, thus forming the distinct “alp horn” shape of the effect of frequency on response latency in auditory lexical decision data.

As in the accuracy model, we can also observe how Trial, Duration, PhonND, and logFreqCOCA interact with NthSession. The effect of Duration appears to be largely stable, persisting in later sessions as well. The effect of PhonND shows a distinction between very low PhonND words and words with medium-low PhonND in earlier sessions (observe the 800 ms contour line) which appears to be lost in later sessions. Otherwise, the effect of PhonND appears to be stable, although overshadowed by NthSession. The effect of logFreqCOCA that distinguishes between low-frequency words on one hand and mid and high-frequency words on the other (800 ms contour line) appears to be somewhat diminished in later sessions. Lastly, the effect of Trial appears negligible in comparison to the effect of NthSession and largely remains stable across sessions.

**Fig. 5:**
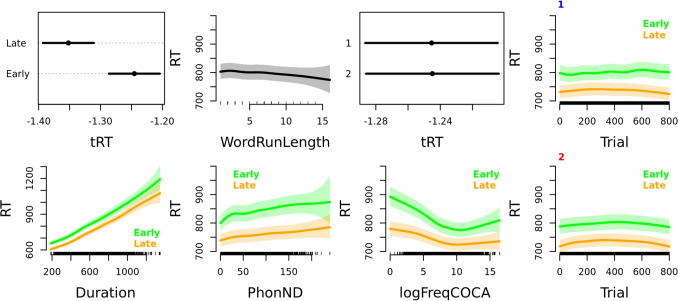
Effects plots of variables for the GAM model fit for response latency in early versus late sessions. In parametric effect plots (plot one of row one showing the effect of NthSessionCat and plot three of row one showing the effect of NthSessionOfDay), the estimates plotted on the *x*-axis are transformed response latency (tRT), while predictor levels are given on the *y*-axis. All other plots are smooth main effect plots, in which the estimate plotted on the *y*-axis is back-transformed response latency (RT), while the continuous predictor is given on the *x*-axis. Data from early sessions is given in *green* and data from late sessions is given in *orange*. Main effects of Trial are presented in the fourth column, separately for the first (*marked with a blue number 1*) and the second (*marked with a red number 2*) NthSessionOfDay. Note that the range of the presented RT values is set to the same range except in the case of Duration. Detailed plot interpretation is given in the text

The presented model clearly indicates that the participants are faster in later versus earlier sessions. However, subtle changes in predictor effects can be masked by the strong effect of NthSession. Therefore, we once more conduct an analysis comparing performance in early versus late sessions and excluding NthPseudowordSet as a predictor (Table 4). The first plot of row one in Fig. 5 also indicates that response latency is shorter in later sessions. The effects of the control predictors WordRunLength and NthSessionOfDay remain the same when the analyses are restricted to the 32 retained sessions.

The smoothed effect of Trial is again presented separately for the first NthSessionOfDay (plot four of row one) and for the second NthSessionOfDay (plot four of row two) and separately for early (green) versus late (orange) sessions. The participants slow down in the first 200 trials and then become faster as they progress through a session. Interestingly, an exception to this is found in the first NthSessionOfDay in the early sessions, as participant performance is largely stable across the session. In the first plot of row three, we see that the effect of Duration is strong and equal both in early and late sessions. The effect of PhonND appears to remain the same in early versus late sessions, except that there is a steeper slope visible for the lowest PhonND values that is lost in later sessions. Lastly, the effect of logFreqCOCA in later sessions is a slightly attenuated version of the effect of this variable in early sessions: the reduction in the slope of the effect is especially visible for low to mid-frequency words.

## General discussion

The first goal of the present paper was to present SingleMALD, a freely available fully crossed auditory lexical decision database for the English language. Large-scale studies such as SingleMALD are valuable resources in their own right, but can provide even richer source of information if combined with other large-scale studies. In that regard, SingleMALD complements the existing large-scale studies using the auditory lexical decision task in the English language that are not fully crossed, such as MALD (Tucker et al., 2019) and AELP (Goh et al., 2020). SingleMALD also offers the possibility of cross-language comparisons if used in concert with BALDEY (Ernestus & Cutler, 2015) or MEGALEX (Ferrand et al., 2018), or comparisons between modalities if used alongside visual lexical decision megastudies (Balota et al., 2007; Keuleers et al., 2012).

The second goal of the present paper was to investigate how participant performance in the auditory lexical decision task changes with extensive testing. Our results show that SingleMALD participant performance changes in terms of their response latency and accuracy, but that the effects of certain stimulus characteristics (duration, phonological neighborhood density, and frequency) largely remain stable across sessions. Note that a comparable statistical model applied to MALD1.1 (i.e., not-fully crossed) data in a separate analysis yielded very similar effects where these three variables are concerned; the similarity is especially high with the SingleMALD sessions we labeled as ‘early’ in the present report. One interesting novelty in SingleMALD data is that extremely high frequency words are responded to with lower accuracy and longer response latency than words with very high frequency. An asymptotic relationship between word frequency and response latency has been recorded before (see Ferrand et al., 2018; Gordon, 1985), but we are not aware of studies that show that extremely high frequency values can be detrimental for lexical decision performance. It might be that these effects are recorded only now because SingleMALD offers a sufficient number of extremely high frequency word trials completed by experienced participants in the auditory modality; a statistical analysis that models non-linear relationships between variables is likewise required to spot this effect. Future work may look into this phenomenon in more detail to provide an explanation.

Although SingleMALD participant performance was fairly stable across sessions, we still found evidence that their strategy and/or the process of spoken word recognition show a degree of change as participants progress through the numerous experimental sessions. Although not large, these changes should be carefully scrutinized. Interestingly, the changes observed for phonological neighborhood density and frequency are not equally present in the entire range of values of these variables.

For example, extensive testing decreased participant response accuracy only when responding to mid-frequency or high-frequency words, but reduced the response latency for low-frequency words (in comparison to middle-frequency words). Both of these changes slightly diminish the effect of frequency on measures of participant performance. One cause for somewhat lower word frequency impact on listener performance could be higher reliance on structural and acoustic cues that betray the lexical status of the stimulus. Keuleers et al. (2010) warn against pseudoword characteristics being different from word characteristics. These differences can be used as a cue that can facilitate lexical decisions and reduce the necessity to rely on lexical characteristics (or other top-down effects).

Similarly, Hargreaves and Pexman (2012) state that participants “implicitly track systematic trends in structural properties of items in order to optimize decision-making in the [visual] LDT” (p. 1). In spoken word recognition, even if pseudowords are made to match the words well, differences between these two kinds of stimuli are difficult to eliminate entirely, simply because the recorded speaker has much less experience with producing pseudowords than words. Listeners likely learn to adapt not only to the structural cues on the item level, but also to the overarching subsegmental, phonetic cues specific to the production of words vs. pseudowords for the recorded speaker. Ferrand et al. (2018) attempt to avoid this by using a computer-synthesized voice, but this procedure may have an impact on ecological validity of the findings. Our participants have a clear signal from a speaker that becomes quite well known to them and respond in the context where they know that word frequency is a less reliable cue whether a certain word would be presented or not, leading to a lower reliance on word frequency and a higher reliance on the bottom-up acoustic signal (see also Hargreaves & Pexman, 2012, for a discussion). However, this explanation does not fully answer the question why practice would differently affect frequency effects at different word-frequency values. Additionally, these results warn against relying solely on linear relationships between variables when larger-scale datasets are available.

The SingleMALD dataset can be used to inform and further develop computational models of spoken word recognition. Models should aim to account for the now established non-linear effects of log frequency in lexical decision (see, e.g., Figure 2 in Brysbaert et al., 2018) that have been already recorded in, e.g., MALD (Kelley & Tucker, 2022b; Nenadić et al., 2024) and MEGALEX (Ferrand et al., 2018). More importantly for the present work, a further challenge would be to simulate changes in listener performance that happen as sessions pass. Crucially, it is an open question to what extent such changes are associated with the speech perception and lexical access processes (expressed, e.g., through weighted connections between units at different levels in a neural network) versus participant decision-making processes. In either case, the model would need to have the ability to keep track of previous trials and adapt to them, while thus far most simulations treated each presentation as isolated (though see Heitmeier et al., 2023). Kapnoula et al. (2024) recently demonstrated the need to account for the plasticity of the process of spoken word recognition at multiple timescales, including short-term plasticity. SingleMALD, alongside other large-scale auditory lexical decision studies, offers data that can be used to design dynamic models of spoken word recognition.

Changes in participant performance could in part be attributed to the fact that pseudowords were, unlike words, repeated in later sessions. This violates the often-held assumption that pseudowords are phonotactically licit word-like speech signals that have a frequency of 0, i.e., do not occur in a listener’s experience. However, Hendrix and Sun (2021) show that (written) pseudoword frequency can be larger than 0. That said, hearing a pseudoword repeated in the same context and by the same speaker may further influence performance, despite the fact that no meaning was explicitly associated with the stimulus during its previous presentation (though it is likely that meaning is never truly eliminated from pseudoword processing, see Chuang et al., 2021; Kelley & Tucker, 2022a). For example, a facilitatory effect of pseudoword repetition priming is observed in the auditory lexical decision task, even when the speaker is not the same or in noisy listening conditions (the delay between repetitions was set to approximately 30 s; Orfanidou et al., 2011).

We acknowledge that the data collected in sessions in which pseudowords were repeated may not generalize well to the typical experimental design where pseudowords are presented only once. Whether repetition effects can extend over weeks of sessions and among thousands of pseudoword recordings is, to the best of our knowledge, an open question. For now, we can only confidently claim that the changes in performance we observed cannot be reduced to pseudoword repetition effects. In our analyses, we found that the variable marking the repetition of pseudowords was excluded from the statistical models as insignificant. This variable was very highly correlated with the number of sessions the participant completed, as repetitions only started happening later in the course of the experiment. Therefore, the number of completed sessions engulfed the effect of pseudoword repetition and offered additional predictive power, indicating changes in performance predicted by the number of completed sessions that are independent of pseudoword repetition. The same can be observed when inspecting changes in participant performance across the first two dozen sessions: although substantial changes in accuracy and response latency are noted, no stimuli repetition is present. Generally, we know little about pseudoword processing in the auditory domain as pseudoword data is often excluded from analysis (for a detailed discussion see Kelley & Tucker, 2022a). More research is necessary to understand the effects of pseudoword processing and specifically the repetition of pseudowords over a longer period of time.

The present findings related to practice effects in the auditory lexical decision task come from a single study using recordings in a single language from a single speaker. In turn, inter-talker variability is high (see, e.g., Kleinschmidt, 2019; Magnuson et al., 2021) and listeners improve their performance after being exposed to, e.g., particular speakers or speaker groups (see, e.g., Bradlow & Bent, 2008; Eisner & McQueen, 2005; Xie et al., 2021). It is still an open question whether different patterns in listener performance would be observed in other large-scale studies where the same participant is extensively tested, such as BALDEY (Ernestus & Cutler, 2015) or MEGALEX (Ferrand et al., 2018), or if the participants were exposed to recordings made by several speakers, as was done in AELP (Goh et al., 2020). From the perspective of a particular participant, BALDEY and AELP are significantly shorter than SingleMALD and it would be important to see whether the same patterns of participant performance change are observed: this may offer insight into whether any changes in performance are based on the absolute number of trials a participant was exposed to, or whether the participants adjust their efforts relative to the number of sessions they are expected to complete. In MEGALEX, participants are exposed to computer-synthesized speech and this circumstance may impact the speed of adjusting to the idiosyncrasies of the recordings. However, if such analyses were to be performed on BALDEY and MEGALEX data, one would need to be aware that these studies included a minimum requirement of participant performance and provided feedback to their participants about their performance.

Certain SingleMALD participants in general, as well as certain participants in a few sessions, exhibited low performance. In the present paper, we used the overall accuracy rate below 60% as a threshold for exclusion. A small number of sessions also diverged from the designed structure of the experiment, for example, by being completed after two sessions were completed already that day by the same participant or by including a word list and pseudoword list combination that was not presented to other participants. We excluded these participants and sessions from our present analyses, but the SingleMALD dataset is freely available and independent researchers are welcome to devise their own criteria for data exclusion. Additionally, it is important to remember that responses collected in an experiment are not independent. Our recommendation is that all of the SingleMALD session data can be used (as, e.g., the frequency effect is still maintained, albeit slightly attenuated, even in the latest sessions) with the inclusion of the session number as a covariate or predictor in statistical analyses (as, e.g., response latency becomes substantially shorter in later sessions). Clearly, independent researchers are welcome to explore how the effects of various predictors change across sessions and devise their own cut-offs accordingly.

In the present report, we stressed the distinction between studies favoring many responses from a small number of participants and studies favoring few responses from a large number of participants. However, with the increase of technological and other resources (such as online testing or crowdsourcing; see Keuleers & Balota, 2015) we expect to see more and more data sets that contain both a large number of participants and a large number of their individual trials and sessions, as was to a degree achieved in MEGALEX (Ferrand et al., 2018). Therefore, we need to control and assess the characteristics of data collected through repeated testing by developing both methodological and analytical (statistical) procedures to account for potential causes of between-session differences.

In SingleMALD, we attempted to account for a number of factors methodologically, e.g., by having the listeners tested in a stable laboratory environment – which may not be possible in the case of online testing – and by having listeners always participate at the same time each day they are tested. Another methodological control that is sometimes applied is explicit performance feedback that can be accompanied with explicit performance requirements (Ferrand et al., 2018; Goh et al., 2020; Keuleers et al., 2010). We argue that the effects of presence or absence of feedback and participant exclusion thresholds in large-scale (auditory) lexical decision experiments and other psycholinguistic tasks are not yet fully explored (see, e.g., goal-setting theory as one framework that problematizes the relationship between goal difficulty, task difficulty, and performance; Locke & Latham, 2002; 2013; Lunenburg, 2011).

We also note that sometimes it may not even be possible to exert strict control or the control thresholds can be difficult to decide upon: for example, what performance level should an L2 speaker achieve to be included in the study. Where methodological control is lacking (or would lead to unknown effects), statistical control plays an important role. Statistical analyses of multi-session data should include as predictors or covariates contextual characteristics. In the present analyses, we considered session number and session number of the day, but others come to mind as well, such as the number of days since previous session or the day of the week when the subject participated. It is largely unknown how these variables can affect participant performance and further explorations may be needed. Importantly, we show using SingleMALD data that the effects of repeated testing or contextual characteristics may not always be linearly related to performance measures.

Despite all of these concerns, the specific characteristics of data collected across a large number of trials and sessions should not be treated as somehow “non-standard”. Data collected from non-experienced participants performing a particular task a small number of times is not the “default” or “control” condition as that data is also shaped by the experience – or lack thereof – that these participants had with the task. One could even argue that we are more likely to tap into realistic language processes when observing humans performing a task with the ease and confidence that comes with extensive experience, as humans do in everyday language use. Large, rich, and carefully constructed data sets – which we aimed for SingleMALD to be – can help us in describing, explaining, and predicting language processing.

## Data Availability

The data are available in the Open Science Framework repository at https://osf.io/wutjv/. Stimuli recordings and other relevant Massive Auditory Lexical Decision project material can be found at https://nascl.rc.nau.edu/resources/massive-auditory-lexical-decision/.

## Appendix Model fit tables for all models

**Table 1:** The generalized additive mixed-effects model fit for response accuracy across all sessions

A. parametric coefficients	Estimate	Std. Error	*t*-value	*p* value
(Intercept)	2.205	0.079	27.950	< 0.0001
NthSessionOfDay2	-0.151	0.007	-23.012	< 0.0001
B. smooth terms	edf	Ref.df	*F*-value	*p* value
s(Trial,Subject)	113.482	323.000	369.379	< 0.0001
s(NthSession,Subject)	252.124	323.000	5879.220	< 0.0001
s(List)	56.030	66.000	502.216	< 0.0001
s(NthSession)	5.131	5.619	76.588	< 0.0001
s(Trial):NthSessionOfDay1	3.896	4.758	77.437	< 0.0001
s(Trial):NthSessionOfDay2	1.009	1.014	26.692	< 0.0001
s(WordRunLength)	1.049	1.097	148.401	< 0.0001
s(Duration)	7.852	8.615	1925.038	< 0.0001
s(PhonND)	5.729	6.763	1101.296	< 0.0001
s(logFreqCOCA)	8.608	8.944	40395.903	< 0.0001
ti(Trial,NthSession):NthSessionOfDay1	4.214	5.536	24.371	0.0004
ti(Trial,NthSession):NthSessionOfDay2	1.019	1.037	0.007	0.9749
ti(Duration,NthSession)	6.231	8.119	112.971	< 0.0001
ti(PhonND,NthSession)	5.730	7.737	52.528	< 0.0001
ti(logFreqCOCA,NthSession)	10.186	11.957	884.592	< 0.0001

Predictors that are marked with “s” represent smoothed predictors and those marked with “ti” represent a smooth for an interaction

**Table 2:** The generalized additive mixed-effects model fit for response accuracy comparing 16 early and 16 later sessions

A. parametric coefficients	Estimate	Std. Error	*t*-value	*p* value
(Intercept)	2.439	0.0797	30.602	< 0.0001
NthSessionCatLate	-0.424	0.011	-40.249	< 0.0001
NthSessionOfDay2	-0.134	0.010	-13.990	< 0.0001
B. smooth terms	edf	Ref.df	*F*-value	*p* value
s(Trial,Subject)	91.959	323.000	15160.443	< 0.0001
s(List)	59.283	66.000	538.191	< 0.0001
s(Trial):Int1.Early	1.377	1.649	38.815	< 0.0001
s(Trial):Int2.Early	1.001	1.001	13.028	0.0003
s(Trial):Int1.Late	1.001	1.002	28.401	< 0.0001
s(Trial):Int2.Late	1.003	1.005	13.480	0.0002
s(WordRunLength)	1.005	1.011	65.290	< 0.0001
s(Duration):NthSessionCatEarly	5.585	6.708	635.652	< 0.0001
s(Duration):NthSessionCatLate	6.028	7.170	339.136	< 0.0001
s(PhonND):NthSessionCatEarly	4.695	5.687	390.951	< 0.0001
s(PhonND):NthSessionCatLate	3.094	3.844	159.179	< 0.0001
s(logFreqCOCA):NthSessionCatEarly	7.753	8.526	11880.763	< 0.0001
s(logFreqCOCA):NthSessionCatLate	7.600	8.428	7503.905	< 0.0001

Predictors that are marked with “s” represent smoothed predictors. “Int” stands for the interaction between NthSessionOfDay with values 1 or 2 and NthSessionCat with values early or late

**Table 3:** The generalized additive mixed-effects model fit for response latency across all sessions

A. parametric coefficients	Estimate	Std. Error	*t*-value	*p* value
(Intercept)	-1.301	0.022	-59.990	< 0.0001
NthPseudowordSet	0.009	0.001	7.955	< 0.0001
NthSessionOfDay2	-0.008	0.001	-14.599	< 0.0001
B. smooth terms	edf	Ref.df	*F*-value	*p* value
s(Trial,Subject)	250.053	323.000	15.001	< 0.0001
s(NthSession,Subject)	295.938	323.000	508.373	< 0.0001
s(List)	61.596	66.000	19.944	< 0.0001
s(NthSession)	8.700	8.735	118.963	< 0.0001
s(Trial):NthSessionOfDay1	7.441	8.044	3.950	0.0002
s(Trial):NthSessionOfDay2	8.259	8.654	16.012	< 0.0001
s(WordRunLength)	5.707	6.584	28.386	< 0.0001
s(Duration)	8.340	8.860	9649.610	< 0.0001
s(PhonND)	7.128	8.009	280.703	< 0.0001
s(logFreqCOCA)	7.933	8.651	1596.153	< 0.0001
ti(Trial,NthSession):NthSessionOfDay1	12.734	14.706	13.637	< 0.0001
ti(Trial,NthSession):NthSessionOfDay2	12.224	14.447	7.521	< 0.0001
ti(Duration,NthSession)	6.485	8.094	36.201	< 0.0001
ti(PhonND,NthSession)	7.089	8.900	17.861	< 0.0001
ti(logFreqCOCA,NthSession)	7.723	9.877	55.885	< 0.0001

Predictors that are marked with “s” represent smoothed predictors and those marked with “ti” represent a smooth for an interaction

**Table 4:** The generalized additive mixed-effects model fit for response latency comparing 16 early and 16 later sessions

A. parametric coefficients	Estimate	Std. Error	*t*-value	*p* value
(Intercept)	-1.233	0.021	-59.257	< 0.0001
NthSessionCatLate	-0.119	0.001	-155.205	< 0.0001
NthSessionOfDay2	-0.007	0.001	-9.026	< 0.0001
B. smooth terms	edf	Ref.df	*F*-value	*p* value
s(Trial,Subject)	238.063	323.000	755.708	< 0.0001
s(List)	65.153	66.000	60.411	< 0.0001
s(Trial):Int1.Early	7.553	8.406	2.959	0.0012
s(Trial):Int2.Early	4.485	5.339	5.909	< 0.0001
s(Trial):Int1.Late	4.538	5.405	5.834	< 0.0001
s(Trial):Int2.Late	5.885	6.929	11.394	< 0.0001
s(WordRunLength)	4.845	5.632	11.303	< 0.0001
s(Duration):NthSessionCatEarly	6.734	7.799	2426.002	< 0.0001
s(Duration):NthSessionCatLate	7.185	8.163	2687.486	< 0.0001
s(PhonND):NthSessionCatEarly	6.497	7.440	121.350	< 0.0001
s(PhonND):NthSessionCatLate	4.378	5.319	46.401	< 0.0001
s(logFreqCOCA):NthSessionCatEarly	6.929	7.930	595.644	< 0.0001
s(logFreqCOCA):NthSessionCatLate	5.914	7.001	266.485	< 0.0001

Predictors that are marked with “s” represent smoothed predictors. “Int” stands for the interaction between NthSessionOfDay with values 1 or 2 and NthSessionCat with values early or late

## Code availability

The analysis scripts and resulting models are available in the Open Science Framework repository at https://osf.io/wutjv/.

## Declarations

### Open Practices Statement

The data and analysis scripts are available in the Open Science Framework repository at https://osf.io/wutjv/. Stimuli recordings and other relevant Massive Auditory Lexical Decision project material can be found at https://nascl.rc.nau.edu/resources/massive-auditory-lexical-decision/.
